# Pomegranate Flower Extract—The Health-Promoting Properties Optimized by Application of the Box–Behnken Design

**DOI:** 10.3390/molecules27196616

**Published:** 2022-10-05

**Authors:** Anna Gościniak, Aleksandra Bazan-Woźniak, Robert Pietrzak, Judyta Cielecka-Piontek

**Affiliations:** 1 Department of Pharmacognosy, Poznan University of Medical Sciences, Rokietnicka 3, 60-806 Poznan, Poland; 2 Faculty of Chemistry, Adam Mickiewicz University in Poznań, Uniwersytetu Poznańskiego 8, 61-614 Poznan, Poland

**Keywords:** pomegranate flowers, design of experiment, inhibition of enzymes, anthocyanins, diabetes mellitus

## Abstract

Herbal raw materials with antidiabetic activity can be a valuable support to therapy. An optimized extraction process allows for the best possible health-promoting effect. Box–Behnken design was employed to optimize the content of methanol used in the extraction mixture, its time, and temperature. The aim of this study was to enhance the efficiency of the pomegranate flowers extraction process in order to obtain extracts with the highest enzyme inhibition power (α-amylase and α-glucosidase), which is important for the antidiabetic effect and the highest antioxidant activity (DPPH assay). In the Box–Behnken design model, the content of pelargonidin-3,5-glucoside–anthocyanin compound that is associated with antidiabetic activity was also optimized as a variable associated with the action profile of pomegranate flower extracts. The process optimization carried out in this study provides a basis for further research using the pomegranate flower extract with the most potent desired properties, essential for supporting diabetes treatment based on pomegranate flowers.

## 1. Introduction

Type 2 diabetes is a disease that affects an estimated 422 million people worldwide [1]. Phytotherapy for diabetes is gaining importance due to its safety, low number of side effects, and multi-directional effects. As a result, scientific interest is growing and there is increasing scientific evidence of the effectiveness of plant raw materials [2]. One of the promising raw material are flowers of pomegranate (*Punica granatum*). In numerous studies, the effect of pomegranate fruit is assessed [3]. However, the activity of other parts, such as roots, tree bark, fruit juice, leaves, and flowers, is less known. Due to the presence of pomegranate flowers in Unani and Ayurvedic medicines for the treatment of diabetes, it is an interesting raw material for research [4]. Huang et al. and Yuhao et al. [4] demonstrated activating effect of pomegranate flower extract on PPAR-gamma receptor–regulators of lipid and glucose metabolism and evaluated the antioxidant and hepatoprotective activity of pomegranate flowers. Kaur et al. [5] evaluated the antioxidant and hepatoprotective activities of pomegranate flowers and confirmed them. Furthermore, Jianjun et al. [6] confirmed the anti-inflammatory effect of the pomegranate flower through its ability to modulate the synthesis of several mediators and cytokines. However, none of these studies focused on extraction optimization. Scientific evidence demonstrates that, in the case of raw plant materials, extraction optimization can produce extracts with the best properties and the highest content of active compounds [7,8,9]. Pomegranate flower activity is associated with the presence of anthocyanins. As the main compounds of this group present in the pomegranate flower, Zhang et al. [10] identified two: pelargonidin-3,5-diglucoside and pelargonidin-3-glucoside.

Response surface methodology (RSM) is a statistical method used for process optimization. The main advantage of this method is to minimize reagent consumption and obtain the best extract on the basis of a small number of samples. One method of RSM is the Box–Behnken design. In this design, each factor or independent variable is assigned one of three equally distributed values, coded −1, 0, +1, and placed at the midpoints of the edges of the process space and at its center [11]. The method of extraction has a significant effect on the biological activity of the plant extracts obtained [12,13]. The choice of suitable solvent and extraction conditions can strongly influence the compounds obtained. Although increasing attention is being paid to solvent-free extraction such as supercritical fluid extraction, the use of organic solvents still remains a common and cheap method. It is therefore worth trying to optimize such extraction to maximize its potential. An interesting discussion on conventional extraction methods was undertaken in their review paper by Mojzer et al. [14] highlighting how conditions such as time and temperature, among others, affect the extraction of polyphenols.

The aim of this study was to apply response surface methodology using the Box–Behnken design to obtain extract with the best antidiabetic and antioxidant properties as well as high content of pelargonidin-3,5-glucoside–anthocyanin compound that is associated with antidiabetic activity.

## 2. Results

### 2.1. Active Compound Content

Effects of methanol concentration (X_1_), temperature (X_2_) and time extraction (X_3_), as in the Pelargonidin-3,5-glucoside content, can be described by the following equation.

Pelargonidin-3,5-diglucoside content = 1.131 + 0.01640 X_1_ + 0.0402 X_2_ − 0.0008 X_3_ − 0.000274 X_1_^2^ − 0.000744 X_2_^2^ − 0.000782 X_3_^2^ + 0.000008 X_1_X_2_ − 0.000342 X_1_X_3_ + 0.000921 X_2_X_3_ (1)

**Figure 1:**
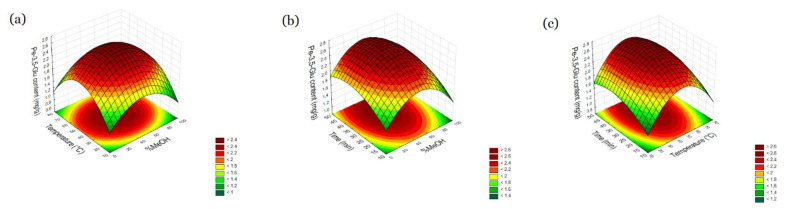
Response surface and contour plots percent of Pel-3,5-Glu content as a function of methanol concentration (X_1_) and temperature (X_2_) (**a**), methanol concentration (X_1)_ and time (X_3_) (**b**), temperature (X_2_) and time (X_3_) (**c**).

Based on the result, it was demonstrated that methanol content and temperature have a significant influence on the content of pelargonidin-3,5-glucoside in the extract (*p* < 0.05). The influence of time is not significant (*p* > 0.05) (Table 1). These results were visualized by using surface plots, which graphically represent the relationships expressed in the abovementioned equation (Figure 1). The extraction efficiency of anthocyanins increases with increasing temperature and reaches its highest value as it was demonstrated that methanol content and temperature have a significant influence on the content of pelargonidin-3,5-glucoside in the extract. Then it decreases again as the temperature increases. A likely explanation of the influence of temperature is the poor stability of anthocyanins in increasing temperature, whereas the short time of extraction was not sufficient to support their recovery [15]. Optimization results show that the best parameter for high content of pelargonidin-3,5-glucoside is 56.21% methanol content. Rocha et al. [16] found similar findings in their study in the case of anthocyanin extraction from raspberry fruit, where also aqueous-alcohol solvent is the most appropriate. Cacace et al. [17] showed that aqueous–alcohol solvent escrowing is most effective for anthocyanin extraction. In their study, the most favorable effect was achieved at a concentration of 60% of ethanol. The effectiveness of anthocyanin extraction with methanol can be increased by adding different proportions of water, which is also supported by a study by Sultana et al. [12] Pelargonidin-3,5-glucoside was chosen for optimization due to the sensitivity of the active compound to extraction conditions and the biological activity of the compound. The pelargonidin-3,5-glucoside content was also confirmed in pomegranate flowers by Zhang et al. [10]. Pelargonidin-3,5-glucoside is also found in red petals of Korean edible rose (*Rosa hybrida*) but its content is lower (31.2 ± 1.4/100 g). A more common constituent is pelargonidin-3-glucoside, which is present in strawberry fruit and is probably responsible for its anti-inflammatory effect [18,19]. This monoglucoside is also found in pomegranate flowers, but the amount is much smaller, and determination was not the aim of this study.

### 2.2. Antidiabetic Activity

In order to evaluate the antidiabetic effect, the inhibitory activities of enzymes responsible for the degradation of polysaccharides were tested. Inhibition of those activities prevents the absorption of polysaccharides and a rapid increase in blood sugar. It is worth noting that the extracts showed a significant possibility of inhibiting the activity of both tested enzymes. The tested concentration that inhibited acarbose from 29.86% to 98.46% was 0.6 mg/mL while acarbose—a substance used as an antidiabetic drug—inhibits 50% of the glucosidase activity at a concentration of 3.43 mg/mL. Similarly, the inhibitory activity on α-amylase is quite strong but, in this case, weaker than acarbose which inhibits 50% amylase activity in a concentration of 6.76 mg/mL, while a concentration of 12.5 mg/mL was used for the study. There is information in the literature on enzyme inhibition relevant to diabetes, but the results are mixed. Purmono et al. [20] showed an inhibitory effect of *Urena lobata* leaf extract on α-amylase and α-glucosidase; however, this effect was 40 and 1000 times weaker than acarbose, respectively. In contrast, Sip et al. [21] showed that aqueous extracts of *Corni fructus* showed significant α-glucosidase inhibitory activity which was 30 times more potent than the acarbose standard. Ji et al. also confirmed the antidiabetic effect of bilberry extract—IC_50_ of α-glucosidase inhibition was 0.31 ± 0.02 mg/mL, which was 13 times lower than that of acarbose (4.11 ± 0.15 mg/mL). The raw material is also rich in anthocyanins, confirming that these compounds are important in the anti-diabetic effect. The anti-diabetic effect of anthocyanins confirms the study by Zhang et al. [22]. Moreover, recent reports show that the synergistic effect of acarbose and polyphenol-rich plant extracts is promising [23]. The study by Kam et al. [24] also finds the effectiveness of pomegranate flower extracts in inhibiting α-amylase and αglucosidase. However, optimization of the extraction process was not used in that study. In our study effects of temperature, methanol concentration, and extraction time on enzyme inhibition were tested and can be described by the following equation.

α-amylase inhibition = 26.16735 + 0.32790 X_1_ − 0.05304 X_2_ + 3.70630 X_3_ − 0.00761 X_1_^2^ 0.00454 X_2_^2^ − 0.05979X_3_^2^ + 0.00172 X_1_X_2_ − 0.00619 X_1_X_3_ + 0.0023 X_2_X_3_ (2)

α-glucosidase inhibition = 15.14492 + 0.98240 X_1_ − 0.03937 X_2_+ 3.52910 X_3_ − 0.01142 X_1_^2^ − 0.003937X_2_^2^ − 0.04876X_3_^2^ + 0.00454 X_1_X_2_ − 0.000829 X_1_X_3_ + 0.00237 X_2_X_3_ (3)

**Figure 2:**
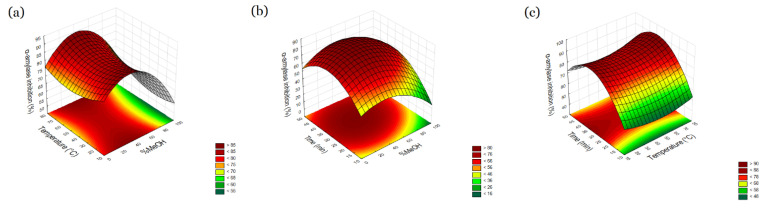
Response surface and contour plots percent of α-amylase inhibition as a function of methanol concentration (X_1)_ and temperature (X_2_) (**a**), methanol concentration (X_1)_ and time (X_3_) (**b**), temperature (X_2_) and time (X_3_) (**c**).

**Figure 3:**
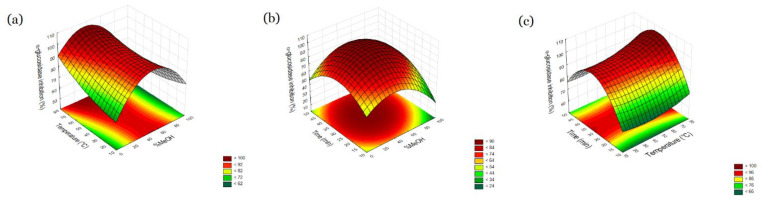
Response surface and contour plots percent of α-glucosidase inhibition as a function of methanol concentration (X_1)_ and temperature (X_2_) (**a**), methanol concentration (X_1_) and time (X_3_) (**b**), temperature (X_2_) and time (X_3_) (**c**).

Based on the result (Table 1) it was demonstrated that methanol content and extraction time have a significant influence on the content of obtained extract (*p* > 0.05), which is responsible for the best possibility of enzyme inhibition significant for antidiabetic activity. Extraction temperature has no significant influence on the content and the result of antidiabetic activity. Analyzing the plot surfaces (Figure 2 and Figure 3) it can be observed that antidiabetic activity increase with increasing time extraction. It was noticed that the highest activities were at 34 min and 32 min for α-amylase and α-glucosidase inhibition, respectively. Then they decreased; however, the decline is moderate. What is more, the temperature is not significant. Therefore, it can be thought that not only anthocyanins which are not stable in temperature are responsible for the antidiabetic effect of the raw material. Considering the dependence of the lack of influence of temperature on the possibility of inhibiting α-amylase and α-glucosidase, it can be suggested that the observed inhibitions are also the result of the presence of compounds from other groups of secondary metabolites. One of the groups of compounds to which the inhibition of enzymes involved in polysaccharide metabolism is attributed is polyphenols [25]. Therefore, as part of the research, the total content of polyphenols in the extracts was determined and treated as a variable in the model.

Apart from the anthocyanin content, pomegranate flowers also contain a high content of flavonoids and tannins, exhibiting antidiabetic potential [26,27]. The efficiency of the extraction of total polyphenols determined by the method according to Kikowska et al. Study [28] is affected by time as well as temperature and methanol content (Supplementary materials, Table S2). Polyphenol content increases with extraction time, and the most favorable temperature is about 60 °C during extraction with 52% methanol. However, for antidiabetic activity, a shorter extraction time is more appropriate (about 35 min). A characteristic property of plant raw materials is their complexity and richness in active compounds. Their joint action can be responsible for the biological effect. The most relevant methanol concentration is 40.10% for α-amylase and 49.30% for α-glucosidase inhibition. For both enzymes the values are similar. This solvent composition is most suitable for the extraction of the active ingredients responsible for the antidiabetic action. Water, due to its higher polarity, is a better solvent for polar compounds; however, compounds of lower polarity will be better extracted by solvents of lower polarity, such as methanol. Methanol was found to be more effective in extracting lower molecular weight polyphenols [29]. Wijngraard and Brunon [30] demonstrated that ethanol, because of its different molecular structure, is more effective in extracting compounds such as chlorogenic acid and flavonoids, and the most effective is a combination of alcohol and water. Moreover, the water content can influence the extraction of active compounds other than polyphenols, which also exhibit biological activity. [31]. In the case of plant raw materials, due to their complexity, it is uncertain which solvent will be the most efficient. Research has shown that in many cases, the most effective solvent is a combination of aqueous and alcoholic solvents, but each raw material must be considered individually.

### 2.3. Antioxidant Activity

Antioxidant activity has a crucial role in preventing the development of diabetes as well as its complications. Diabetes leads to depletion of the cellular antioxidant defense system and is associated with increased production of free radicals. Oxidative stress can exacerbate insulin resistance and beta-cell dysfunction [32]. Hyperglycemia has been found to promote low-density lipoprotein (LDL) lipid peroxidation leading to free radical production [33]. Elevated sugar levels result in the formation of advanced glycation end-products (AGEs), which are responsible for increased oxidative stress, among other things. As a result of elevated sugar levels, glycation end products (AGEs) are formed, which are responsible, inter alia, for causing the formation of free radicals [34]. Oxidative stress can also lead to inflammation by activating various biochemical pathways. The consequence is the formation of microangiopathies. Damage to the structure of the capillaries or arteries leads to impaired transport of nutrients, disruption of the body’s defenses, and, consequently, to complications such as diabetic foot [35]. Based on scientific evidence, antioxidants have a beneficial effect in the treatment of diabetes.

The antioxidant activity of the tested extracts expressed as IC_50_ ranges from 0.333 mg/mL to 0.964 mg/mL, while the value for ascorbic acid is 0.0085 mg/mL. This activity is lower than the reference substance but comparable to the antioxidant activity of herbal antidiabetic blends [36]. Kaur et al. [37] have shown that pomegranate flower extract exhibits strong antioxidant activity, which also affects hepatoprotective activity. However, the antioxidant activity of flower extracts from other species may be much stronger. The activity of the methanolic extract of damask rose (*Rosa damascena*) was up to four times more potent than ascorbic acid [38]. However, the DPPH free radical scavenging activity of methanolic extracts of *Hibiscus sabdariffa* showed weaker activity than ascorbic acid [39]. Raw materials rich in anthocyanins are also berries which, as Bae et al. [40] showed, also have a strong ability to scavenge the DPPH radical.

**Figure 4:**
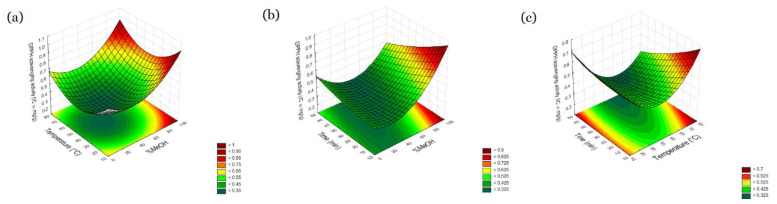
Response surface and contour plots percent of antioxidant activity as a function of methanol concentration (X_1)_ and temperature (X_2_) (**a**), methanol concentration (X_1)_ and time (X_3_) (**b**), temperature (X_2_) and time (X_3_) (**c**).

Antioxidant activity was mainly affected by methanol concentration. The influence of temperature and time is not significant. Antioxidant activity increase with methanol. Concentration up to a maximum of approximately 34.56% and then decreased with a further increase in methanol concentration. By observing the fitting curves (Figure 4), it can be observed that the antioxidant activity increases up to a temperature of about 50 °C and then decreases, and the antioxidant activity increases with time. However, these relationships are not statistically significant (*p* > 0.05). Antioxidant activity can be related to the content of total polyphenols, where similar relationships can be observed (Supplementary Material, Figure S1). Polyphenols are a broad group of compounds that exhibit strong antioxidant properties

DPPH = 0.625895 − 0.002733 X_1_ − 0.013582 X_2_ − 005766 X_3_ − 0.000095 X_1_^2^ − 0.000196 X_2_^2^ − 0.000073 X_3_^2^ − 0.000001 X_1_X_2_ − 0.000126 X_1_X_3_ − 0.000154 X_2_X_3_ (4)

### 2.4. Analysis of the Influence of Variables on the Properties of the Extract

The effect of linear, quadratic, or interaction coefficients on the response was tested for significance by analysis of variance. Based on the F-value and *p*-value, the significance of the factors was evaluated, and the results are shown in Table 1. Based on the results presented, it can be concluded that the content of pelargonidin-3,5-glucoside is significantly influenced by methanol content and temperature. However, in the case of enzyme inhibition, time and methanol content are important, and the temperature of the reaction is not significant. In contrast, for antioxidant activity, only the methanol content is most relevant. Fan et al. showed similar relationships in optimizing the extraction of anthocyanins from sweet potatoes where the extraction effect strongly depended on temperature, and extraction time did not have as much influence. Fan et al. [41] showed similar relationships in optimizing the extraction of anthocyanins from sweet potatoes where the extraction effect strongly depended on temperature, and extraction time did not have as much influence. In the study by Zou et al. [42], the effect of methanol content and temperature and also the ratio of solid to liquid which was not taken into account in our study proved to be significant. In this study, it was investigated that the time after reaching equilibrium did not affect the extraction efficiency, which also confirms the results of our study. To date, there have been no studies on optimizing the extraction process to obtain the strongest enzyme inhibition; however, Bungthong et al. [43] proved in their paper that extraction time has a significant effect on the inhibition of glucosidase and amylase by silk proteins

### 2.5. Model Fitting

The experiments were designed according to the Box–Behnken design, and the results are presented in Table 2. There was a close agreement between experimental and observed values. The R^2^ and R^2^_adj_ values for the proposed models have high values. For Pel-3,5-Glu content R^2^ = 0.90 R^2^_adj_ = 0.71; for α-glucosidase and α-amylase inhibition R^2^ = 0.93 R^2^_adj_ = 0.81 and R^2^ = 0.93 R^2^_adj_ = 0.79, respectively. For antioxidant activity optimization model values reach R^2^ = 0.92 and R^2^ = 0.70. These values demonstrate good model fit and applicability.

### 2.6. Validation of the Models

In order to confirm the validity of the model, the experimental values are compared with the predicted values at the optimal points of extraction (Table 3). The results showed that the values obtained experimentally are close to those determined by the model. This indicates the sufficiently good fit of the model and its usefulness.

## 3. Materials and Methods

### 3.1. Chemicals and Reagents

Standard compounds used in the HPLC analysis, including pelargonidin-3,5-glucoside (≥95.0%) were supplied by Sigma-Aldrich, St. Louis, MO, USA.

Reagents used in the biological activity studies, including α-D-glucopyranoside (PNPG), α-glucosidase from *Saccharomyces cerevisiae* (Type I, lyophilized powder, ≥10 units/mg protein), acarbose, 2,2-diphenyl-1-picrylhydrazyl, DPPH (2,2-diphenyl-1-picrylhydrazyl) were supplied by Sigma-Aldrich, St. Louis, MO, USA, HPLC grade acetonitrile and methanol were obtained from Merck (Warsaw, Poland). High-quality pure water and ultra-high-quality pure water were prepared by using a Direct-Q 3 UV Merck Millipore purification system (Merck, Darmstadt, Germany).

### 3.2. Plant material

Plant raw material, pomegranate flowers, was purchased from NANGA (Złotów, Poland), lot number: 1/9/2020/O, Country: Albania. The product comes from conventional cultivation.

### 3.3. Extract Preparation

First, 1.0 g of dry pomegranate flower was ground in a mortar and placed in a conical flask. Extraction was carried out with 10 mL of a suitable solvent acidified with 0.01% HCOOH, at a temperature and time according to the experimental design as described in the following Section 3.4. Extracts were made up to a volume of 10 mL and stored feezed at −20 °C. 

### 3.4. Response Surface

A Box–Behnken statistical screening design was used to statistically develop the model and to investigate and evaluate the main, interaction, and quadratic effects of process parameters (temperature, time, organic solvent content) on antioxidant activity, antidiabetic activity, and polyphenol content. Box–Behnken design (BBD) was used to minimize the number of extracts obtained and to evaluate the relevant factors. The calculations and analyses were performed using the program Statistica 13.3 (TIBCO Software Inc, Palo Alto, CA, USA). The variables X1–3 are coded according to Table 4.

The suggested model to which the experimental data were fitted is a second-order polynomial model. The following equation was applied:

(5) $$Y=b_{0}+\sum b_{i}X_{i}+\sum b_{ii}X_{ii}^{2}+\sum b_{ij}X_{i}X_{j}$$

where *Y* represents the response variable (pelargonidin-3,5-content, α-glucosidase inhibition, α-amylase inhibition, and antioxidant activity); *X_i_* and *X_j_* are the independent variables (temperature, methanol concentration, and extraction time); β_0_, β_i_, β_ii_, and β_ij_ are the regression coefficients for intercept, linear, quadratic, and interaction coefficient, respectively.

### 3.5. HPLC Analysis

**Figure 5:**
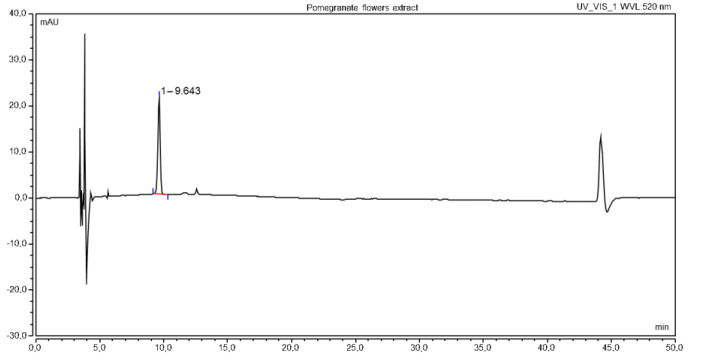
The chromatogram of pomegranate flowers extract.

High-performance liquid chromatographic methods, using HPLC-DAD (Dionex Thermoline Fisher Scientific, Waltham, MA, USA) with gradient elution, were developed to determine anthocyanin 3,5-*O*-glucosides of pelargonidin. The HPLC gradient method, coupled with the DAD detector, allowed the qualitative and quantitative determination of pelargonidin-3,5-glucoside in extracts from pomegranate flowers. The determinations were carried out using a LiChrospher RP18-5 4.6 mm × 250 mm, 5 µm; the mobile phase contained 5% formic acid in water(A) and 5% formic acid in methanol (B). The gradient developed for the needs of the method assumed changes in the mobile phase according to the scheme: 0–15 min B = 15–55%, 15–20 min B = 55–65%, 20–30 min B = 65–70%, 30–40 min B = 70–75%. The method considers 10 min re-equilibration time to determine the phase equilibrium column relative to the initial injection phase. The phase flow was set to level 1 mL/min, injection for all test samples was 30 μL, and the detection was carried out at 520 nm. The chromatogram of the extract is shown in Figure 5.

### 3.6. Antioxidant Activity

A total of 25.0 µL of the test sample and 175.0 µL of DPPH radical solution (3.9 mg/50 mL methanol) were applied to the 96-well plate. The control sample was a mixture of the DPPH radical solution and the solvent used in the extraction. The blank was the absorbance value obtained after mixing 25.0 µL of the extraction solvent and 175.0 µL of the solvent used to dissolve the DPPH radical (methanol). The sample blanks were the absorbance of 25.0 µL of extract at the appropriate concentration and 175 µL DPPH solvent. Six repetitions were carried out. The plate was shaken for 5 min at 25 °C and then incubated for 25 min at room temperature. Absorbance was measured at 517 nm.

The free radical scavenging capacity (%) of the test extracts and the standard substance (ascorbic acid) was calculated from equilibrium:

(6) $$DPPH_{scavenging\;activity}\left(\%\right)=\frac{A_{0}-A_{1}}{A_{0}}\times 100\%$$

where *A*_0_ is the absorbance of the control, and *A*_1_ is the absorbance of the sample. From the results obtained, the IC50 value was calculated, which corresponds to the concentration of extract required to inhibit 50% of the activity.

### 3.7. α-Glucosidase Inhibitory Assay

Briefly, 50 μL of sample solution (50 μg/mL), 50 μL of 0. 1 M phosphate buffer (pH 6.8), and 30 μL of α-glucosidase solution (0.5 U/mL) were preincubated in 96-well plates at 37 °C for 15 min. Then, 20 μL of a 5 mM solution of *p*-nitrophenyl-α-D-glucopyranoside (pNPG) in 0.1 M phosphate buffer (pH 6.8) was added and incubated at 37 °C for 20 min. The reaction was terminated by adding 100 µL of sodium carbonate (0.2 M) to the mixture. The absorbance of the released *p*-nitrophenol was measured at 405 nm. The absorbance of the sample without extract was used as a control. The absorbance in the absence of the enzyme was used as a blank control. The degree of enzyme inhibition, expressed as percentage inhibition, was calculated according to the following formula:

$$Enzyme\;Inhibition\;\left(\%\right)=\frac{A_{0}-A_{1}}{A_{0}}\times 100\%$$

where *A*_0_ is the absorbance of the control reduced by the sample background (100% enzyme activity), and *A*_1_ is the absorbance of the tested sample reduced by the sample background.

### 3.8. α-Amylase Inhibitory Assay

The amylase inhibition test was performed according to the methodology described by Studzińska-Sroka et al. [36] as 20 µL of α-amylase (2.0 U/mL) and 20 µL of the test extract (12.5 mg/mL) were preincubated in a 96-well plate at 37 °C. After 20 min, 20 µL of previously prepared warm 0.1 M phosphate buffer (pH 6.9) and 0.5% starch solution was added to the wells and re-incubated for 20 min at 37 °C. Then, 60 µL of color reagent containing 96 mM 3,5-dinitrosalicylic acid solution (20 mL), 5.31 M potassium sodium tartrate solution in 2 M sodium hydroxide (8 mL), and deionized water (12 mL) were added to each well. The plate was incubated at 85 °C for 15 min, then cooled to room temperature, and 80 µL of water was added. Absorbance was measured at 540 nm (Multiskan GO 1510, Thermo Fisher Scientific, Vantaa, Finland). The absorbance of the enzyme solution without extracts was used as a control for total enzyme activity. The absorbance in the absence of the enzyme was used as a blank control. The degree of enzyme inhibition was expressed as a percentage of inhibition and calculated according to the following formula:

$$Enzyme\;Inhibition\;\left(\%\right)=\frac{A_{0}-A_{1}}{A_{0}}\times 100\%$$

where *A*_0_ is the absorbance of the control reduced by the sample background (100% enzyme activity), and *A*_1_ is the absorbance of the tested sample reduced by the sample background.

## 4. Conclusions

Pomegranate flowers show antidiabetic and antioxidant potential and can be a raw material useful in supporting diabetes treatment. It appears that the anthocyanin content is mostly influenced by the process temperature and the methanol content of the extraction mixture. Pomegranate flowers contain 2.296 mg/g of pelargonidin-3,5-glucoside, the presence of which is associated with the biological activity of the raw material, under the optimized conditions. The raw material exhibits potent antidiabetic activity similar to that of acarbose tested in glucosidase and amylase inhibition models. A concentration of 0.6 mg/mL inhibits α-glucosidase in a range of 29.86% to 98.46%, depending on the extraction conditions used. It also has the ability to almost completely inhibit amylase at a concentration of 12.5 mg/mL. Strong antioxidant activity is also important. The application of optimization methods allows for the preparation of extracts with the best properties while ensuring lower solvent and energy consumption. In the following work, the RSM method was applied using the Box–Behnken method. Due to the good fit of the model, it can be concluded that it may be used to evaluate the effects. It has been shown that depending on the assessed output effect the significance of the influence of particular components varies (the content of methanol, temperature, and time extraction). This is influenced by factors such as the stability of anthocyanins, the total amount of phenols extracted from the raw material, and the fact that the biological effect of extracts is influenced not only by individual components but also by the phenomenon of synergism occurring between the substances present in the raw material. However, the optimal parameters for each of the evaluated parameters are close, and choosing the corresponding ones is possible.

## Figures and Tables

**Table 1:** The effect of linear, quadratic or interaction coefficients on the response.

Source	DF	Sum of Squares	F-Value	*p*-Value
Pelargonidin-3,5-Glucoside content				
X_1_	1	0.013	0.321	0.596
X_2_	1	0.001	0.018	0.898
X_3_	1	0.110	2.748	0.158
X_1_X_1_	1	0.895	22.455	0.005 *
X_2_X_2_	1	0.411	10.321	0.024 *
X_3_X_3_	1	0.059	1.477	0.278
X_1_X_2_	1	0.00010	0.005	0.945
X_1_X_3_	1	0.136	3.414	0.124
X_2_X_3_	1	0.246	6.168	0.556
α-amylase inhibition				
X_1_	1	577.656	12.886	0.016 *
X_2_	1	5.462	0.121	0.741
X_3_	1	509.656	11.369	0.002 *
X_1_X_1_	1	1336.406	29.811	0.003 *
X_2_X_2_	1	55.629	0.662	0.453
X_3_X_3_	1	668.260	14.907	0.012 *
X_1_X_2_	1	18.498	0.412	0.549
X_1_X_3_	1	86.239	1.923	0.224
X_2_X_3_	1	2.991	0.066	0.806
Error	5	44,828		
α-glucosidase inhibition				
X_1_	1	1435.346	16.733	0.009 *
X_2_	1	9.891	0.115	0.748
X_3_	1	1098.641	12.807	0.016 *
X_1_X_1_	1	2191.982	26.006	0.004 *
X_2_X_2_	1	170.780	1.522	0.272
X_3_X_3_	1	433.849	5.058	0.074
X_1_X_2_	1	24.573	0.286	0.615
X_1_X_3_	1	3.427	0.039	0.849
X_2_X_3_	1	1.489	0.017	0.900
Error	5			
Antioxidant activity				
X_1_	1	0.172	15.868	0.010 *
X_2_	1	0.002	0.176	0.691
X_3_	1	0.017	1.59	0.261
X_1_X_1_	1	0.194	19.203	0.007 *
X_2_X_2_	1	0.054	5.099	0.074
X_3_X_3_	1	0.001	0.091	0.775
X_1_X_2_	1	0.000	0.001	0.973
X_1_X_3_	1	0.036	3.284	0.129
X_2_X_3_	1	0.013	1.236	0.317
Error	5	0.011		

* statistically significant value (*p* < 0.05).

**Table 2:** The Box–Behnken design for the three independent variables. Experimental and predicted response for α-glucosidase and α-amylase inhibition, antioxidant activity (DPPH) and Pelargonidin-3,5-glucoside content. ^a^ the average value of the results obtained.

Run	x _ 1 _	x _ 2 _	x _ 3 _	α-glucosidase Inhibition [%]		α-amylase Inhibition (%)		Antioxidant Activity DPPH (IC_50_ mg/g)		Pelargonidin-3,5-glucoside Content [mg/g]	
				Experimental ^a^	Predicted	Experimental ^a^	Predicted	Experimental ^a^	Predicted	Experimental ^a^	Predicted
E1	−1	−1	0	97.24	94.51	79.53	76.13	0.510	0.579	1.637	1.463
E2	1	−1	0	65.43	62.76	60.28	54.84	0.964	0.876	1.141	1.372
E3	−1	1	0	84.66	87.33	68.04	73.49	0.463	0.551	1.741	1.51
E4	1	1	0	62.76	65.49	57.39	60.79	0.910	0.841	1.205	1.378
E5	−1	0	−1	54.74	61.49	53.56	55.18	0.511	0.411	1.589	1.869
E6	1	0	−1	29.86	36.54	25.23	28.90	0.837	0.894	1.368	1.244
E7	−1	0	1	93.46	86.77	65.53	61.86	0.563	0.507	1.558	1.682
E8	1	0	1	64.88	58.13	55.77	54.15	0.511	0.611	2.364	2.084
E9	0	−1	−1	85.28	81.26	62.17	63.94	0.463	0.495	2.217	2.11
E10	0	1	−1	87.24	77.82	70.93	63.86	0.567	0.579	1.496	1.446
E11	0	−1	1	94.07	103.49	71.11	78.17	0.529	0.517	1.695	1.745
E12	0	1	1	98.46	102.48	83.33	81.56	0.402	0.370	2.355	2.462
E13	0	0	0	94.07	96.16	83.99	82.50	0.352	0.352	2.584	2.581
E14	0	0	0	98.46	96.16	81.84	82.50	0.369	0.352	2.535	2.581
E15	0	0	0	95.94	96.16	81.68	82.50	0.333	0.352	2.626	2.581

**Table 3:** Predicted and experimental results.

Investigated Effect	Predicted Values	Experimental Value
Amylase inhibition (%)	84.57	87.82 ± 1.66
Glucosidase inhibition (%)	95.86	95.37 ± 2.15
DPPH_sc_ (IC_50_)	0.297	0.320 ± 0.010
Pg-3,5-Glu content (mg/g)	2.620	2.296 ± 0.026

**Table 4:** Levels and code of variable chosen for Box–Behnken design.

Variable, Unit	Symbol	Level		
	x	Low Level (−1)	Center Level (0)	High Level (+1)
Methanol content [%]	*X*_1_	0	50	100
Extraction temperature [°C]	*X_2_*	20	45	70
Extraction duration of time [min]	*X_3_*	15	30	45

## Data Availability

The data is contained within the article or Supplementary Material.

## Supplementary Materials

The following supporting information can be downloaded at: https://www.mdpi.com/article/10.3390/molecules27196616/s1. Table S1. BBD with the observed responses and predicted values for TPC; Table S2. ANOVA for response surface models: estimated regression model of relationship between response variable (TPC) and independent variables (X_1_,X_2_,X_3_) from pomegranate flowers; Figure S1: Response surface and contour plots TPC as a function of X_1_ and X_3_ (a), X_1_ and X_2_ (b), X_2_ and X_3_.

## Sample Availability

Samples of the compounds are available from the authors.
