# Supplementary material for: Pomegranate Flower Extract—The Health-Promoting Properties Optimized by Application of the Box–Behnken Design
Source: Molecules. 2022 Oct 5;27(19):6616. doi: 10.3390/molecules27196616 (PMC9573694; doi:10.3390/molecules27196616)
Supplement: Supplementary file 1 [file molecules-27-06616-s001.zip › molecules-1932935-supplementary.pdf]

Supplementary Materials

Table S1. BBD with the observed responses and predicted values for TPC.

Run	x_1	x_2	x_3	Total Polyphenolic Content [mg GAE/g]	
				Experimental	Predicted
E1	-1	-1	0	32,91	30,25
E2	1	-1	0	25,63	25,8
E3	-1	1	0	32,14	31,97
E4	1	1	0	26,9	29,57
E5	-1	0	-1	32,88	36,91
E6	1	0	-1	25,4	26,59
E7	-1	0	1	41,47	40,27
E8	1	0	1	47,77	43,74
E9	0	-1	-1	55,75	54,38
E10	0	1	-1	42,19	38,32
E11	0	-1	1	41,96	45,83
E12	0	1	1	66,01	67,38
E13	0	0	0	63,82	62,7
E14	0	0	0	63,76	62,7
E15	0	0	0	60,52	62,7

Table S2. ANOVA for response surface models: estimated regression model of relationship between response variable (TPC) and independent variables (X1,X2,X3) from pomegranate flowers.

Source	DF	Sum of Squeres	F-Value	P-Value
**TPC**				
Model	9	3070,051	18,29	0,003*
X_1	1	23,457	1,296	0,306
X_2	1	15,072	0,834	0,403
X_3	1	210,197	11,616	0,019*
X_1X_1	1	2118,192	117,0519	0,0001*
X_2X_2	1	323,023	17,85	0,008*
X_3X_3	1	12,895	14,91	0,437
X_1X_2	1	1,051	0,058	0,819
X_1X3	1	47,497	2,625	0,166
X_2X_2	1	90,481	19,539	0,007

Equation S1 Equation representing the quadratic model fit determination of the sum of polyphenols in pomegranate flowers

$$TPC\ [mg\ GAE/g] = 31{,}61783 + 0{,}76753\ X_1 + 0{,}62915\ X_2 – 0{,}51789\ X_3 – 0{,}00958X_1^2 – 0{,}01497\ X_2^2 - 0{,}00831\ X_3^2 – 0{,}00041\ X_1X_2 - 0{,}00459\ X_1X_3 + 0{,}02507\ X_2X_3$$

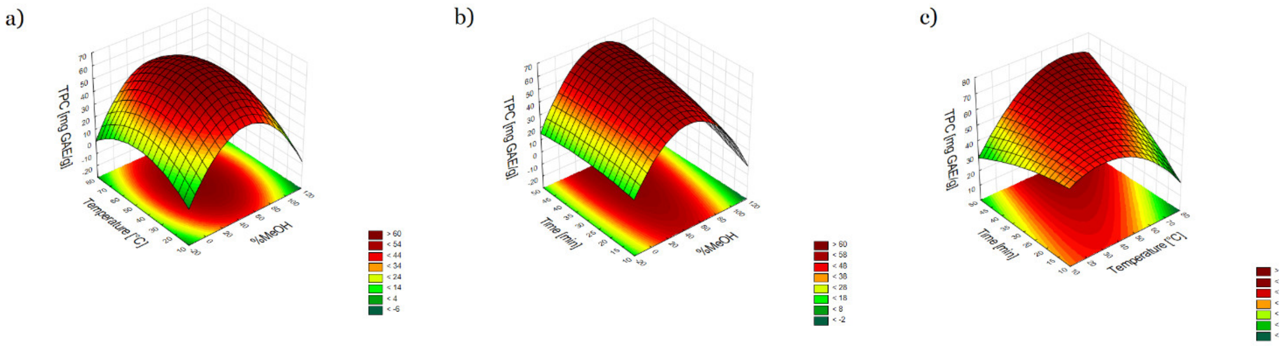

Figure S1 Response surface and contour plots TPC as a function of X_1 and X_3 (a), X_1 and X_2 (b), X_2 and X_3.

Interpretation

Based on the results, there is a good model fit (p < 0.05). The polyphenol content of the raw material is influenced by all the study factors - methanol content , temperature and time. The highest amount of polyphenols is obtained during extraction with 55% methanol at 53 degrees for 43 min.

Methods

The determination of total polyphenols was performed according to the methodology of Kikowska et al. To 25 μL of extracts or gallic acid solution (in the concentration range of 6.25-100 μg/mL), 200 μL of distilled water, 15 μL of Folin-Ciocalteu reagent, and 60 μL of 20% sodium carbonate solution were added. Under dark conditions, the plate was shaken for 5 min at room temperature at 600 rpm and then incubated for another 25 min. Five replicates were used for analysis. The total gallic acid content of the extracts obtained was estimated using the calibration curve of the standard substance and presented in milligrams of gallic acid equivalents (GAE) per 1 g of plant material.
